# How Do We Perceive a Humorous Manager? Manager Humor, Impression Management, and Employee Willingness to Work With the Manager

**DOI:** 10.3389/fpsyg.2021.628350

**Published:** 2021-09-13

**Authors:** Yael Brender-Ilan, Abira Reizer

**Affiliations:** ^1^Department of Economics and Business Management, Ariel University, Ariel, Israel; ^2^Department of Behavioral Sciences, Ariel University, Ariel, Israel

**Keywords:** humor, impression management, computer-mediated communication, behavioral intentions, gender differences

## Abstract

Humor is a form of communication that is intended to be entertaining and produce positive affective and cognitive responses from receivers. Nonetheless, humor in the workplace is a complicated matter. It has been recognized as a valuable tool for managers because it can activate various favorable outcomes and alter employees’ perception of the manager’s warmth and competence (impression management), but not always to the benefit of the manager. In our studies, the use of humor showed changed attitudes toward a manager’s warmth and competence, and eventually influenced the employee’s behavioral intentions. In Study 1, we tested the use of managerial humor in two emails. The humorous manager was perceived as warm, but not competent. Impression management mediated the employee’s willingness to work with the manager. In Study 2, we tested the use of managerial humor with one introductory email. In this study, we also monitored the gender of both the manager and the employee. Once again, the humorous manager was perceived as warm and humor mediated employees’ behavioral intentions. As for competence, gender moderated the results, such that male employees perceived humorous female managers as more competent, while female employees perceived humorous male managers as less competent. Practical implications are presented.

## Introduction

Humor has been recognized as a valuable tool for managers because it can activate several favorable outcomes in the fields of business and organizational behavior (Duncan et al., 1990; Mesmer-Magnus et al., 2012). Organizational scholars refer to humor as an effective tactic and managerial resource, particularly under limited tangible resource allocation (Robert et al., 2016; Cooper C.D. et al., 2018).

Over the past two decades, there has been a growing interest in managerial and leadership’s use of humor, and their potential impact in shaping employees’ attitudes and behaviors (Kong et al., 2019), and business unit effectiveness (Priest and Swain, 2002; Cooper K.M. et al., 2018). Specifically, researchers found that positive humor used by managers during their interactions with their employees increased organizational and employee creativity (Csikszentmihalyi, 1996; Li et al., 2019); employee positive emotions and work engagement (Goswami et al., 2016); employees’ psychological capital (Li et al., 2019); manager-subordinate relations (Messmer, 2012; Liu et al., 2019); and leadership effectiveness ratings (Decker and Rotondo, 2001). In addition, managers’ positive use of humor also serves to facilitate a friendly workplace environment (Pandita et al., 2019).

Despite the increasing interest in the impact of humor, the theoretical understanding of the use of humor among managers is incomplete, and the process through which it affects employees’ outcomes has not been fully identified) (Karakowsky et al., 2019). Therefore “[perhaps out of all communication strategies used by leaders, the use of humor may be the most promising, but it is also the least understood” (Crawford, 1994, p. 54). This fundamental quest remains unresolved even 20 years later (Martin et al., 2003; Veselka et al., 2010; Yam et al., 2018).

There have been several gaps in the extant literature as well as unanswered questions related to managerial use of humor. First, while most of the published studies focused on managerial humor as a trait, there is a lack of research when it comes to examining actual managerial humor expression (Kong et al., 2019). Specifically, Kong et al. (2019), in their recent extensive meta-analytic review on managerial humor, concluded that leader humor expression is a better predictor of employee behaviors and attitudes than leader humor as a trait. They call for further exploration of actual leader humor expression.

In addition, while most of the studies up till now have focused on cross-sectional and correlational designs (Kong et al., 2019), there is a lack of research findings on managerial leadership humor expression. Finally, there is an inadequate understanding of the processes by which managerial humor expression impacts employees’ perceptions and behavioral intentions. Moreover, there is also a need to deepen our understanding regarding the potential moderators and mediators of these relationships.

The current study addresses Kong et al. (2019) call by focusing on several unexamined goals in the managerial humor literature. Our study aims to: (1) empirically investigate the contribution of leader humor expression on employees’ behavioral intentions (willingness to work with the manager); and (2) explore the mediators and moderators that explain the associations between leadership humor expression and behavioral intentions. As humor may impact impression management processes (Bitterly and Schweitzer, 2019), we will also examine whether employees’ impression management perceptions regarding their manager may mediate the associations between managerial humor expression and employees’ intentions to work with their manager. As for the third goal: (3) Since leadership humor expression may be perceived differently depending on the leader’s gender (Evans et al., 2019), and as gender was presented as a potential moderator for future examination by several scholars (Mesmer-Magnus et al., 2018; Kong et al., 2019), we will also investigate the moderating role of managerial gender. Finally, (4) while most, if not all, previous measurements in the literature on managerial humor have been based on employees’ and managers’ self-report, which are potential sources of social desirability and common method biases (e.g., Antonakis, 2017; Kong et al., 2019), we will further examine the research hypotheses by using an experimental design. Additionally, up until now all existing managerial humor expression research has pertained to face-to-face (hereafter FtF) interactions, but not all manager-employee interactions are FtF. While previous work suggested that managers who use humor are perceived more favorably by their employees (i.e., Messmer, 2012), this research did not address written communication, but primarily FtF interactions that were measured via employee’ self-report. This is a critical gap in the available research because FtF interactions provide a very different medium for communication immediacy than computer-mediated communication (hereafter CMC) contexts (Schulze et al., 2017). Our final research goal is to be not only methodologically novel, but also theoretically innovative. The current study will address and use the most recent and common workplace communication channel – email correspondence (Machili et al., 2019). In addition, the current work is the first to examine managers’ humorous expression in email communication. Our investigation will examine how adding a humorous expression in a manager’s email sent to employees affects employees’ perceived impression of the manager and their subsequent behavioral intentions.

### Computer-Mediated Communication in the Workplace

In this age of globalization, current technology, coupled with the stipulation of immediate communication and quick reactions, directs workplaces to use CMC for day-to-day professional activity. Though FtF is certainly not deemed redundant, these two communication methods require different personal characteristics – the latter being termed a twenty-first century competency one cannot do without (Schulze et al., 2017). Emails have become mainstream in organizational life (Derks and Bakker, 2010). It is fast, inexpensive, accessible, and easy to replicate; thus, it streamlines communication (Brown et al., 2014). Employees seem to prefer email communication and they use it to communicate information as well as emotions (Byron, 2008). Yet, the use of email can lead to miscommunications – a drawback that needs to be closely monitored and improved to avoid interpersonal misunderstandings during organizational written interactions (Byron, 2008). Still, the benefits of this mode of written communication still far outweigh the disadvantages. When employees send emails to colleagues, the recipients can access and respond to the email at their convenience, thus increasing work flexibility and creating a sense of timeliness (Mano and Mesch, 2010). Despite the growth and prominence of mobile messengers and chat apps, email remains an integral part of daily online life. In 2020, the number of global email users stood at four billion, meaning over 50% of the world’s population was using email. This number is projected to reach 4.6 billion users in 2025 (The guardian, 2016; Eurofound and the International Labour Office, 2017; Levin and Kurtzberg, 2020; Statista, 2021).

While FtF communication encompasses different cues, including non-verbal cues, CMC is unique because it is documented, more detailed, and more informative (Tudini and Liddicoat, 2017). Specifically relevant for this study is the difference between types of humorous communications. Humor can be communicated via spoken conversation and written text. However, both are considered verbal humor (Norrick, 1993), as opposed to non-verbal humor emerging from pictures or body language. Importantly, the SIDE model of dehumanization has found different effects on behavior when comparing CMC and FtF interactions (e.g., Spears, 2017; Vilanova et al., 2017), mostly when discussing norms of behavior, which are crucial in workplace environments (Mackey et al., 2021). In this study, we stress the difference between *conversational humor* that can be accompanied by non-verbal cues and elicit certain effects, and *verbal humor* which takes the form of written text, specifically in our case – emails (Dynel, 2009). While there are several ways in which humor and laughter can be elicited, the most common is humor emerging spontaneously from a particular situation (Martin and Kuiper, 1999). This type of humor can occur in the workplace, but cannot be easily and accurately measured, since we, as researchers, are not there to witness and document it. Moreover, the reactions and laughter elicited by conversational humor are psychologically different from textual humor (Martin and Ford, 2018). Importantly, written jokes have been extensively studied, their mechanisms examined and compared (Ruch et al., 1993). The General Theory of Verbal Humor (GTVH) has studied these mechanisms and it was found to be conducive for testing jokes and their translation (Attardo, 2017). In addition, the ideas behind the GTVH could also be used to understand humor and what makes a joke funny (Raskin, 2012), as well as to analyze humor occurring in conversations. Still, these mechanisms are mainly used to analyze the linguistics of written jokes (Attardo, 2017). In this study, we focus on this latter type of humor – “verbal” written humor that is conveyed in emails.

The use of email at work reflects professional demands, individual preferences, and the inclusion of emotions. This use of emotions in emails can create miscommunications which negatively impact organizational communication (Byron, 2008). While boundaries in emails are group dependent (Cecchinato et al., 2015), and can employ varying emoticon communicative functions (Skovholt et al., 2014; Glikson et al., 2018), industry users (compared to academia users) have a better understanding of the boundaries between work and personal usage, indicating that it is the working environment that influences people’s boundary practices (Cecchinato et al., 2015). In addition to the variability in email messaging among environments, culture is also a factor. Email communication styles and content, in terms of individual psychological space (i.e., private life, confidentiality, work orientation, and social distance), vary widely across cultures (Holtbrügge et al., 2013).

As a twenty-first-century skill (Schulze et al., 2017), competent managers are also evaluated by their ability to communicate effectively using emails given that, in general, communicating well with employees has been shown to predict important organizational outcomes, such as individual and team performance and leadership (e.g., Young et al., 2000; De Vries et al., 2010). Consequently, it is vital to study ways in which this type of communication affects employees’ evaluation of managers’ competence.

We regard emails as a type of professional conversation between managers and employees (Tudini and Liddicoat, 2017). However, since emails lack the non-verbal cues FtF conversations possess (Holtbrügge et al., 2013; Schulze et al., 2017), individuals need to use different cues to emphasize and express themselves (e.g., emoticons, unconventional orthography, and non-standard punctuation) (Darics, 2010; Vandergriff, 2013; Kalman and Gergle, 2014). In the current study, we aim to test the effect of humorous discourse framed by the concrete text of an example of a work email. We will study the effects of written humor on the impression/first impression an employee forms regarding a manager/new manager, who uses humor in an email.

### Humor and Managerial Positive Humor Expression

Humor is a form of communication (Smith et al., 2000) that is intended to be entertaining (Cooper, 2005, 2008), and produces positive affective and cognitive responses in receivers (Crawford, 1994; McGraw et al., 2015; Warren and McGraw, 2016). Nonetheless, humor in the workplace is a complicated matter. Positive humor was found to have beneficial effects in the workplace in relation to employees’ well-being, productivity, and managers’ socialization (e.g., Gkorezis et al., 2011; Mak et al., 2012; Smith and Khojasteh, 2014). In a comprehensive literature review and meta-analysis of leader humor, Kong et al. (2019) explain the myriad uses of humor by leaders and its effects on followers, and provide constructive critiques of leader humor research. Specifically, in their extensive meta-analytical review they distinguish between the idea of a managerial humor trait (an individual difference of the manager) and managerial humor expression (behavioral). Although the subject of managerial humor trait received more empirical attention, Kong et al. (2019) concluded that humor expression is a stronger predictor of employees’ attitudes and behavior intentions, and called for further empirical support for the contribution of leadership humor expression.

In the best-case scenario, humor is non-hostile, and affirming of self and others. However, negative comicality as well as self-defeating humor and aggressive humor also exist (Martin et al., 2003). Martin’s approach has also been applied to the organizational setting. It was indicated that humor, which concentrates on the self (for purposes of self-enhancement or self-appreciation), is positively related to personal well-being measures and positive interpersonal communications, while negative humor styles impaired personal well-being, often leading to interpersonal conflicts (e.g., Mesmer-Magnus et al., 2012). When examining the unique contribution of different types of positive humor, it appears that the use of affiliative humor facilitates the advancement and preservation of social support networks, which foster and enhance well-being.

Humor has long been studied as a form of human influence (O’Quin and Aronoff, 1981), while its social functions have been extensively studied from an anthropological perspective (Martineau, 1972). In a recent meta-analysis that examined the effect of humor on persuasion (Walter et al., 2018), researchers found that overall, humor has a significant, but weak, effect on persuasion; some level of influence on knowledge; and only a minor impact on attitudes and behavioral intentions. When it comes to humor in the workplace, the results are more prominent. *Managerial humor expression* is defined as a behavioral construct focusing on the way managers express humor during interpersonal interactions with their employees. It is “a communication tactic used by the leader. the core of which is the leader[’s] sharing of funny events with the employees with the intention to amuse them” (Pundt and Herrmann, 2015, p. 109). A meta-analysis (Mesmer-Magnus et al., 2012) found positive effects for employees’ use of humor (satisfaction, enhanced work performance, workgroup cohesion, coping effectiveness, health, and decreased levels of stress, work withdrawal, and burnout) as well as managers’ use of humor (general satisfaction, workgroup cohesion, satisfaction with supervisor, enhanced subordinate work performance, satisfaction, perception of supervisor performance, satisfaction with supervisor, and workgroup cohesion, along with reduced work withdrawal). Humor was also found to be indicative of employee OCB (Martin et al., 2004; Tremblay et al., 2016), and employees’ happiness, well-being, and short and long-term positive emotional and psychological outcomes (Robert and Wilbanks, 2012; Kim et al., 2016; Wijewardena et al., 2017).

The functional perspective refers to humor expression as a managerial tool. By adding humor to a conversation, the manager can promote the accomplishment of work (Malone, 1980; Newstrom, 2002), re-frame difficulties so that they seem less disturbing, and ease negative emotions (Grugulis, 2002). Managers can use humor as a mode of communication – to transmit ideas (Grice, 1989) or merely entertain their employees (Raskin, 1985; Bitterly and Schweitzer, 2019). Nonetheless, though managerial humor was vastly shown to have positive effects on employees and organizational culture (Holmes and Marra, 2002; Shah, 2018), research also found a potential downside regarding humor at work (Yam et al., 2018). These studies show that humor at work is not necessarily perceived as beneficial in all areas of the world (Yang et al., 2017; Wang et al., 2018). In particular, the disruptive perspective views humor usage as a type of behavior that conflicts with the serious nature of business (Duncan et al., 1990). Moreover, humor might impose norms (Meyer, 2000), generate interpersonal tension, and create an assertion of power over others (Holmes and Marra, 2006).

### Humorous Emails and Willingness to Work With the Manager

Known positive outcomes of leadership communication competence are employee satisfaction, motivation, and commitment (Mikkelson et al., 2015). On the other end of this spectrum, negative outcomes were also found, including stress, job tension, emotional exhaustion, and turnover (Khan et al., 2010; Sun et al., 2019). Malone (1980) concluded that leader humor should be examined and recognized as a tool that enables managers to enhance employees’ satisfaction and productivity. He also suggested that humor may serve as a facilitator that helps leaders get things done (Malone, 1980). It seems that a consensus is emerging, which perceives managerial positive humor as a form of socioemotional exchange currency that can shape, develop, and build manager-employee relations (Cooper K.M. et al., 2018; Kong et al., 2019).

The dynamics of the manager-employee relationship and its effectiveness are influenced by several managerial factors. These include managers’ previous work experience, affiliation scores, and the ability to address both the personal and professional needs of employees by creating a friendly workplace environment (Pandita et al., 2019). Humor can help improve this dynamic, by forming strong friendship networks at work, which has been associated with the willingness to work together. Humor can also contribute to positive affect, which tends to promote improved interpersonal relationships at work (Romero and Cruthirds, 2006; Robert et al., 2016). Moreover, humor moderates the relationship between leadership style and unit-level performance; and the managerial use of humor has been shown to encourage the forming of strong friendship networks, as well as enhanced leader-employee relationship quality (LMX) (Avolio et al., 1999; Liu et al., 2019). While there is some indication that managers who use positive humor expression in day-to-day interactions are perceived favorably by their employees (e.g., Messmer, 2012), little is known regarding the impact of humor in terms of behavioral intentions such as willingness to work with the supervisor. There are three primary theories about humor in the literature, which explain the contribution of managerial humor expression to employee intentions and behaviors. The first is *relief theory* (Freud, 1960), according to which humor is derived from the release of built-up emotions that are otherwise suppressed. Therefore, humor is considered a defense mechanism (Cooper, 2008), a stress-relief tool, which has therapeutic benefits when coping with sensitive and uncomfortable topics (Kuiper, 2012). The second theoretical conceptualization refers to *the positive affective benefits of humor*. Humor can facilitate positive emotions that promote individual cognitions and behaviors (Cooper, 2005). Effective leaders usually manage their employees’ emotions (Cropanzano et al., 2017), and impact the positive evaluative judgment of their employees, thus facilitating employees’ attitudinal and behavioral outcomes. Employees’ positive emotions, triggered by the manager, can also broaden and build employees’ personal resources (Fredrickson, 2013). The third and most popular theory is that of social exchange. According to *social exchange theory* (Blau, 1964), a manager and an employee-follower have mutual interactional episodes that consist of resource exchange (Graen and Uhl-Bien, 1995). Managers are not expected to express humor toward their employees. Therefore, an expression of humor signals managerial supportiveness and friendliness toward employees that extends beyond what is expected (Blau, 1964), in addition to managerial desire to form good relationships with employees (Cooper, 2008). Therefore, managerial expression of humor serves to build and expand the employee’s socioemotional resources. Strong exchange relationships with the manager not only contributes to employees’ affective response toward the job and the organization, it also motivates them to reciprocate the manager’s positive attentions by diligently fulfilling job requirements (Cooper K.M. et al., 2018). Cooper K.M. et al. (2018) integrated the three theories and conceptualized *managerial humor expression* as an interpersonal resource that promotes socio-psychological functioning among employees. When supervisors use positive forms of humor with their subordinates, they send a message that the individual is worthwhile and well liked, which may promote that individual’s self-esteem (Mesmer-Magnus et al., 2018). Indeed, a recent meta-analysis study suggested that humor expression is associated with employee intentional behaviors such as their willingness to remain with the organization (Kong et al., 2019). However, their analysis was based on three studies (Sobral and Islam, 2015; Cooper C.D. et al., 2018; studies 1 and 2), which emphasize the need for further examination of the topic. Following this work, in the current study we expand this perspective by suggesting that humor expression may shape employees’ willingness to work with the manager during their early initial stages of interaction.

Based on these links connecting leader humor expression and positive employees’ behavioral outcomes, we infer that willingness to work with the supervisor will undoubtedly be enhanced by the manager’s use of positive humor. This is because, as was shown, humor has ample positive effects on employees’ positive evaluation of the manager and interaction with the manager. This can translate into one’s willingness to work with this type of positive-effect-creating agent – the manager.

In addition, we expand previous work by focusing on written communication sources.

According to Social Information Processing (SIP) theory (Walther, 1992), participants in CMC settings are probably able to communicate interpersonal information to the same degree as in FtF settings. One way to communicate relational information in the CMC setting is to use humor in the text. Therefore, according to SIP, the use of humor in texts signals relational communication and serves as a potential compensating mechanism to non-verbal channels in CMC conversations (Hancock, 2004). It was recently suggested that humorous text messages may be an effective tool for shaping beneficial organizational image. Shin and Larson (2020) conducted three experimental marketing studies suggesting that humorous responses to a client’s complaints by a service agent have a favorable influence on the firm’s perceived attractiveness, regardless of the type of humor used (i.e., affiliative or aggressive humor) The impact of humorous emails on employees’ willingness to work with managers has not been examined previously, and only a few leadership research studies have examined the contribution of managers’ humorous expression and employees’ behavioral intentions. However, we suggest that employees might positively evaluate written humorous messages sent by their managers via email correspondence. Therefore, these employees are likely to respond positively to their manager’s requests, as was previously suggested in organizational studies.

*Hypothesis 1*: A manager’s expression of humor in an email will increase the employee’s willingness to work with the manager.

### Humor and Impression Management

*Impression management* (hereafter IM) appears to be a strong individual-level variable when considering aspects that are likely to influence the relationship between managerial humor and employee outcomes. In the workplace, effective IM projects the “right” images of an individual’s personal and professional life (Bolino et al., 2016), and is considered a desired managerial quality. Much of the IM literature has focused on two common impressions managers seek to convey: warmth and competence (Holoien and Fiske, 2013; Koslowsky et al., in press).

*Warmth* represents the interpersonal tendency to demonstrate friendliness, communion, helpfulness, and trustworthiness (Cuddy et al., 2011; Fiske et al., 2018). The *competence* dimension captures intrapersonal capabilities such as ability, intelligence, agency, skills, confidence, and resource deployment (Fiske et al., 2007; Cuddy et al., 2011). These two dimensions of warmth and competence can also be described as “liking versus respecting” (Fiske et al., 2007), and they generally account for 82% of the variance in personal impressions in social psychology studies (Wojciszke et al., 1998; Fiske et al., 2007). These unique dimensions of warmth and competence are considered central dimensions in the organizational literature as well (Bitterly and Schweitzer, 2019).

From a theoretical perspective, humor impacts the IM process. The speaker’s use of humor can determine the way the audience perceives the speaker’s intentions and motives, which in turn, fundamentally alters the way the audience evaluates the speaker (Bitterly and Schweitzer, 2019). Indeed, it was indicated that the use of humor shapes IM processes (Cooper, 2005; Stoll, 2015; Bitterly et al., 2017). Humor can increase employees’ positive evaluations of managers as it builds social relationships (Cooper, 2005). Therefore, we assume that managerial use of humorous communication will increase employees’ perceptions of their manager’s warmth. The associations are more complicated in terms of the relationship between humor and competence. In many ways, the decision to use humor can be a very risky business (Bitterly et al., 2017). On one hand, managers who use humor can be perceived as showing lower levels of competence because the use of humor in the workplace is viewed as inappropriate and distracting, and is associated with “playing around,” rather than focusing on tasks in a serious manner (Plester, 2009; Martin and Ford, 2018). People who use humor at work may be perceived as lacking dedication (Taylor and Bain, 2003; Westwood and Johnston, 2013). On the other hand, employees may value managers who are willing to “risk” using humor in their conversations, given that humor can be misunderstood or negatively assessed (McGraw and Warner, 2014; Martin and Ford, 2018). One might assume that the uncertainty is even higher when the joke-teller interacts with an unfamiliar audience and uses a CMC interface as opposed to FtF conversation. Therefore, when a manager decides to use humor, despite all the potential obstacles, the employees might perceive the use humor as demonstrating confidence.

Although there is a lack of research regarding the impact of humorous managerial message on impression management in experimental design, the literature provides some support for the contribution of humorous messages to IM processes in the workplace. For example, Bitterly and Schweitzer (2019) recently indicated that humor increases warmth and competence perceptions among interviewers during face-to-face job interviews. Specifically, they found that job candidates who use humor while disclosing negative information were perceived more favorably – in terms of both warmth and competence by the interviewees – as opposed to job candidates who do not use humor. In addition, studies show that displaying humorous responses to publicly viewable online customer complaints after a service failure can contribute to corporate image perceptions. Specifically, a humorous reply can have a positive influence on the firm’s perceived attractiveness, which was linked to higher perceived firm innovativeness (Shin and Larson, 2020).

Our study is based upon the perception that managerial humor is an effective mechanism for “building friendships in the office and breaking down barriers” (Mesmer-Magnus et al., 2018 p. 704). Managers’ positive humor increased their employees’ tendency to evaluate them as more socially attractive (Wanzer et al., 1996) and more favorable (Mesmer-Magnus et al., 2018). It was also suggested that managerial positive humor expression facilitated employees liking of the leader (Hoption et al., 2013). However, further research is needed to explore the effects of leader humor expression and employee perceptions (Kong et al., 2019). In addition, the current literature does not provide a clear argument for how managerial humor expression is incorporated by employees during early interactions in an experimental design, and how the receiver interprets it during an email correspondence. The goal of the current study is to address these gaps in the literature. Therefore, we hypothesize the following:

*Hypothesis 2a:* The use of humorous messages via email increases employees’ perceptions of managerial competence.

*Hypothesis 2b:* The use of humorous messages via email increases employees’ perceptions of managerial warmth.

### The Mediating Role of IM

In the current study, we examine how managerial humorous messages can increase employees’ positive impression of their manager, ultimately increasing employee willingness to work with the manager. Whereas prior research has focused primarily on the direct impact of humorous managerial messages on employees’ outcomes – suggesting that people benefit from delivering humorous messages because observers respond positively to such messages (Mesmer-Magnus et al., 2012) – relatively little research has examined the mediating mechanisms of these associations. As IM increases one’s chances to form and maintain long-term relationships and friendships (Uziel, 2010), we assume that positive IM will increase employees’ willingness to work with their manager.

Humor is an interpersonal form of communication (Holmes et al., 2019) that signals affection (Hoption et al., 2013) and warmth (Bitterly and Schweitzer, 2019). In addition, positive and funny humorous messages signal high confidence, competence, and power (Bitterly et al., 2017).

The managerial literature suggests that managerial use of humor predicts high-quality manager-employee relationships because employees feel more comfortable at work. This increases their engagement with supervisors (Pundt and Herrmann, 2015) and causes them to view their supervisors as relationship-oriented (Decker and Rotondo, 2001). Specifically, positive humor expression was found to be related to favorable reactions to the leader (Decker, 1991). In addition, managers perceived as higher in humor use were perceived as more likable and more effective in their positions (Rizzo et al., 1999) – all of which can boost employees’ willingness to work with the manager. Managerial favorability mediated the relationship between managerial humor as a personality trait and employee outcomes such as job satisfaction, employee organizational commitment, and employee organizational pride (Mesmer-Magnus et al., 2018). More importantly, leader humor was found to foster employees’ organizational citizenship behavior through the mediating mechanism of leader-member exchange (Cooper K.M. et al., 2018).

Therefore, we assume that the use of humor could also elicit a positive evaluation of managers’ competence skills, as well as increase employee willingness to form a mutual exchange relationship with the manager such as willingness to work with the manager. Finally, IM can occur in many social channels, including FtF as well as written communication (Koslowsky et al., in press). Hence, we assume that FtF theories can be applied to email correspondence as well.

*Hypothesis 3a*: Perceived warmth will mediate the relationship between a managerial humorous message and employees’ intentions to work with the manager.

*Hypothesis 3b*: Perceived competence will mediate the relationship between a managerial humorous message and employees’ intentions to work with the manager.

## Study 1

In Study 1, we examined whether the inclusion of a humorous message in a manager’s email about a work directive impacts the *employee*’s willingness to work with the manager, and whether warmth and competence mediate this linkage. For this purpose, we used an experimental design with three conditions. Participants received two consecutive emails that included instructions for performing a task (organizing and calculating the department’s financial expenses). The treatment group received the basic message with a humorous sentence; the control group received the same message, but the humorous sentence was replaced with a general clarification sentence of the same length; and the neutral group only received the basic message. This methodology allowed us to compare the humorous email to the two other conditions: control and neutral.

### Method

#### Participants

To detect a medium effect size with 80% power (alpha = 0.05), G^∗^Power suggested we would need 90 participants (Faul et al., 2009). Two trained MA research assistants recruited 100 employees using Network respondent-driven sampling (Wejnert and Heckathorn, 2008; Heckathorn, 2011). The final sample included 63% women. The participants’ mean age was *M*_age_ = 31.75 (*SD*_age_ = 11.41); approximately 50% held a BA degree and 7% held an MA degree or higher. The participants’ average weekly work hours were 40.14 (*SD*_hours_ = 12.18), and 63% were in a non-management position. The experiment included three between-subjects conditions: a humor condition (*n* = 33) and two control conditions – a control condition (*n* = 34) and a neutral condition (*n* = 33). Participants were randomly assigned to one of the three conditions.

#### Procedure and Humor Manipulation

The experiment included two steps. The first step was to send a gender-neutral email describing the purpose of the study and the assurance of anonymity of all responses, and a message from a “manager” to the “employee” (the participant) with an assignment (a simple question of choosing an unnecessary departmental expense item). The participant was asked to send the name of the unnecessary expense item via email to the “manager.” Upon receiving the name of the item, the “manager” sent a final email thanking the “employee” and asking him/her to complete an online questionnaire. Participants were informed that upon completion of the final questionnaire, they would automatically be included in a $25 draw.

In order to test the effect of the use of humor by the manager in an email, we used three versions. To provide a consistent and unbiased examination of the treatment effect (Rosenbaum, 1987, 2002), we included a second control group. In this second control group (the neutral group), the participants only received the basic message. All versions included a basic message from the manager asking the participant to remove one unnecessary expense item from a list of departmental expenses (see Appendix for the email wording of the three versions). The treatment group received the basic message and a joke. The study received research ethics committee (institutional review board) approval.

#### Measurements

##### Perceived warmth and competence

To measure perceived warmth and competence, we used items developed by Fiske et al. (2002). *Perceived warmth* was measured using 4 items (sincere, friendly, tolerant, warm). *Perceived competence* was measured using 5 items (skillful, confident, intelligent, competent, and efficient). Participants responded on a 7-point Likert scale – ranging from 1 (totally disagree) to 7 (totally agree). Cronbach’s alphas were (0.90) for perceived warmth and (0.93) for perceived competence.

##### Willingness to work with the manager

To measure behavioral intentions, we used one question: “Would you like to continue working with this manager?” Participants responded on a 5-point Likert scale – ranging from 1 (do not want to) to 5 (definitely want to).

##### Manipulation check

To assess whether the message was perceived as humorous, participants answered the following question: “Did you find the manager’s email amusing?” Participants responded on a 5-point scale – ranging from 1 (not amusing at all) to 5 (very amusing).

### Results

Descriptive statistics and intercorrelations are shown in Table 1.

**TABLE 1:** Descriptive statistics and intercorrelations.

Variable	Mean	*SD*	1	2	3
1. Humor	–	–	–	–	–
2. Warmth	3.51	0.92	0.31**	(0.93)	–
3. Competence	3.57	0.94	0.12	0.69***	(0.90)
4. Behavioral intentions	3.58	0.91	0.09	0.55***	0.58***

*N* = 100. Reliability coefficients are displayed on the diagonal. Warmth = Employees’ perceived warmth of the manager; Competence = Employees’ perceived competence of the manager. Humor 0 = no, 1 = yes; **p < 0.01, and ***p < 0.001.

#### Manipulation Check

An analysis of variance (ANOVA) revealed a significant difference between the experimental and control groups [*F*(2,97) = 22.77, *p* < 0.001)]. *Post hoc* analysis (Scheffe) indicated that the participants in the humor condition group were significantly more amused (*M* = 3.45, *SD* = 1.03) than the participants in either the control group (*M* = 2.00, *SD* = 0.89) or the neutral group (*M* = 2.27, *SD* = 0.88).

#### Examining the Effects of Willingness to Work With the Manager and IM

An analysis of variance (ANOVA) revealed a non-significant difference in willingness to work with the manager among the experimental, neutral, and control groups [*F*(2,97) = 0.46, *p* = 0.64)]. Thus, *H1* was not supported. In order to examine *H2a* and *H2b –* predicting that a humorous comment in an email increases employees’ perceptions about their manager’s warmth and competence – a multivariate analysis of variance (MANOVA) was conducted. Box’s M (11.53) was not significant, *p*(0.08) > (0.05), indicating that there are no significant differences between the covariance matrices. Therefore, the MANOVA assumption is not violated and Wilk’s Lambda is an appropriate test to use. The MANOVA analysis indicated a significant effect *F*(4,192) = 2.97, *p* = 0.02; Wilks’ ∧ = 0.89, partial η^2^ = 0.06. Follow-up univariate ANOVAs indicated that warmth was significantly different for the humor condition group as opposed to the control and neutral groups *F*(2,97) = 5.00, *p* = 0.01, partial η^2^ = 0.09]. The *post hoc* test (Scheffe) indicated that the humor condition group perceived the manager as warmer (*M* = 3.91, *SD* = 0.78), as opposed to the control group (*M* = 3.34, *SD* = 0.93) and the neutral group (*M* = 3.30, *SD* = 0.92), thus supporting *H2a*. However, there was no significant difference among the three groups regarding employees’ perception of their manager’s competence. Thus, *H2b* was not supported.

#### Examining the Mediating Effects of Perceived Competence and Warmth

**FIGURE 1:**
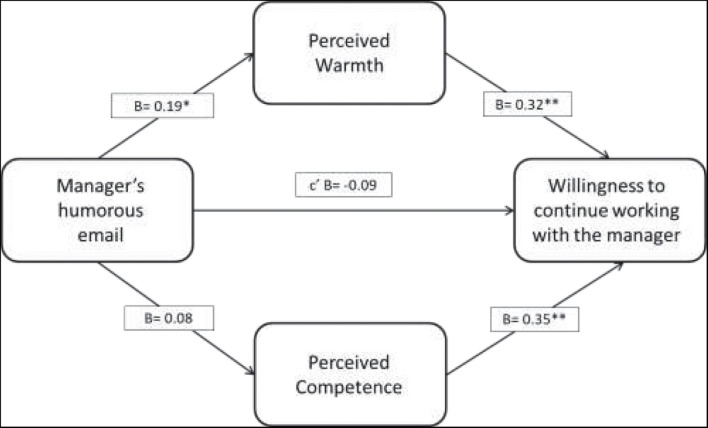
The mediation model for Study 1 with unstandardized coefficients. Both mediators were tested simultaneously (Model 4, Hayes, 2017).

In order to examine the mediating effects of perceived competence and warmth on the impact of the humorous message, and on willingness to continue working with the manager (H3), we adopted Hayes (2017) procedure to test for regression and moderated mediation. We ran the analysis using the SPSS macro PROCESS 3.2. To reduce non-essential multicollinearity concerns (Enders and Tofighi, 2007), independent variables were mean-centered before computing product terms. Since there was no significant difference between the control and neutral conditions, we combined these groups. We conducted a bootstrap procedure (5,000 resamples) to examine the mediating path. The first bootstrap analysis revealed a significant indirect path from receiving a humorous message via perceived warmth to willingness to continue working with the manager [effect = 0.19; 95% CI (0.02, 0.41)]. However, the mediating role of perceived competence on the impact of humor on willingness to continue working with the manager was not significant [effect = 0.08; 95% CI (−0.06, 0.25)]. When controlling for the mediator, there was no direct association between receiving a humorous message and willingness to work with the manager (effect = −0.07, *p* = 0.66), thus indicating a full mediation path. Figure 1 illustrates the findings.

### Discussion

Our findings support the functional perspective in humor that refers to humor as a managerial tool that can further work accomplishments (Newstrom, 2002). Therefore, humor would increase employees’ willingness to work with the manager through the mediating role of warmth perception. In addition, the participants in both the control group and the humor condition group evaluated their managers relatively high, as opposed to the neutral group. The current findings did not support the disruptive perspective that views humorous amusement as contradicting with the serious manner in which business should be conducted (Duncan et al., 1990) and characterizes humorous employees as lacking dedication (Taylor and Bain, 2003; Westwood and Johnston, 2013).

The positive benefits of humor on warmth, and the lack of negative impact in terms of competence, are very encouraging to humor research, providing considerable evidence that people are more sensitive to information that is related to the *warmth* dimension. Research suggests that *warmth* judgments occur prior to *competence* judgments (Willis and Todorov, 2006), and account for a greater portion of the IM process (Abele and Wojciszke, 2007). In addition, warmth is a fundamental aspect of the evolutionary perspective, as it determines our approach-avoidance behavioral reactions to others (Peeters, 2001; Fiske et al., 2007).

Although the study supported our hypotheses, it is not without limitations. First, the study included only 100 participants divided into three groups. Therefore, the lack of support for the mediating role of competence may stem from the relatively small sample size, even though the sample size was supported by power analysis. In addition, previous studies suggested that the manager’s gender (Evans et al., 2019) as well as employees’ gender (Bitterly et al., 2017) should be examined as a potential moderator in the associations between humor and workplace outcomes.

Another important point in this study is that the manager sent an email using gender-neutral wording. Considering the above discussion, we further presumed that the manager’s gender could possibly influence the results. Therefore, the moderating effect of gender needs to be addressed.

## Study 2

As mentioned above, the potential role of gender may affect the humorous exchange between managers and employees. We therefore set three objectives for Study 2. First, we aimed to further test the use of humor in an email that does not initiate employees’ action, but is rather a mundane managerial message. Second, we wished to reproduce and validate the effect of humor on IM that was established in Study 1. Importantly, Study 2 included two new key conditions. One, the sample was considerably larger (265 vs. 100 participants). Two, the email communication was a single short email and did not include further interaction with the manager. This was done in order to assess whether the effect of humor on IM can also be reproduced in a brief communication. Third, in an attempt to add to the theoretical understanding of the nature of the relationship between humor and first impression, we introduced the moderating effect of gender.

### The Moderated Mediation Model: Gender as a Moderator

We chose to use *the humor receiver’s gender* as a moderating variable, since toggling the gender of the joke-teller (Hooper et al., 2016) and the gender of the receivers (Bressler et al., 2006) may profoundly influence the joke’s success. To this end, the current work addresses the recent calls by Bitterly et al. (2017), Kong et al. (2019), and Mesmer-Magnus et al. (2018) to expand current knowledge on the managerial use of humor regarding how the gender of the manager and the audience may affect the success of a humorous message.

Gender differences in humor style usage were extensively studied. Martin et al. (2003) found gender differences in all humor styles, with males reporting higher levels on all styles compared to women. In their meta-analysis, Kong et al. (2019) reported various results of gender differences in humor use, and encouraged the use of leader gender and follower gender as moderators in the study of humor and leadership. Crawford, (2003) studied the origins and psychological mechanism of gender differences in humor. He concluded that women and men use humor to construct their interaction with same-gender and mixed-gender individuals and express their group’s agenda. Over the past decades, women’s humor, in particular, has been affected by the women’s movement and the related political atmosphere.

Gender was also used as a moderator in studies examining the associations linking humor, stress, and outcomes (Abel, 1998). In terms of humor appreciation, Herzog (1999), replicating Mundorf et al. (1988), did not find that males and females have a different appreciation of humor, mostly in relation to opposite-gender victim and sexual humor. However, he did find changes in the appreciation of hostile humor toward female victims, attributing the change and his failed replication to the influence of the women’s movement.

We assume that the receivers’ gender will impact the way humor is perceived (appropriate vs. inappropriate), and therefore will ultimately affect the receivers’ willingness to work with the manager. To further understand the role of gender in the relationship between humor and outcomes, we refer to the experiment of Evans et al. (2019), which shows that using humor increases men’s, but decreases women’s, status, performance, and leadership ratings, in comparison to their same-gender counterparts. Bitterly et al. (2017) found that effective and appropriate humor could increase *status*, and argued that appropriate humor signals high confidence and competence levels. However, in their study humor was manipulated for men only and gender was not fully examined. Since Heilman (2012) found that gender stereotypes can serve as shortcuts for forming impressions, a more thorough examination of gender is required. Expectations regarding the way women and men should behave are widely shared, and are mostly instinctive in individuals’ behavior (Hentschel et al., 2019). Therefore, women’s behavior in the workplace might produce a different effect than a similar behavior when performed by men (e.g., Gupta et al., 2018). Scholars have paid little attention to characteristics of the humor’s source and audience. We maintain that this is an oversight, predominantly when examining the issue of gender. The ideas that male and female behavior will be interpreted differently were expressed 25 years ago (e.g., Crawford and Gressley, 1991) and are still maintained today (Tosun et al., 2018). For example, Cooper K.M. et al. (2018) found some differences in the way male and female students interpreted instructors’ humor (males found it funny, while females found it offensive). Riordan and Glikson (2020) found differences among men’s and women’s reaction to leader’s use of emojis. Female recipients rated a leader using emojis as less effective and, generally found the use of emojis by leaders as less appropriate for the workplace.

Additionally, we rely on notions from the parallel-constraint-satisfaction theory (PCST) of functionality vs. disruptiveness of behaviors, to support the argument that gender stereotypes will affect employees’ impression of managers’ humor differently, depending on the manager’s gender (Read and Simon, 2012). Thus, for male managers, we expect that the effect of humor will be similar to that observed by Evans et al. (2019), meaning – it will be evaluated positively because it is seen as more functional compared to humor expressed by female managers.

It is reasonable to postulate that humor used by female managers will be interpreted differently. Researchers found that female stereotypes include lower expectations regarding rationality, achievement, and agency (Fiske et al., 2002). This could mean that the positive impression extended to men does not apply to women. The above reasoning suggests that gender plays a central role in the relationship between humor and status. In addition, according to Bitterly et al. (2017), when the humor is considered functional it is likely to signal competence. Consequently, we expect that a dysfunctional use of humor will reduce competence ratings and lead to a negative relationship between humor and competence for women.

*Hypothesis 4a:* Managers’ gender moderates the mediating role of competence on the impact of humor on behavioral intentions, such that the mediating role of competence is positive for male managers and negative for female managers.

*Hypothesis 4b:* Receivers’ gender moderates the mediating role of competence on the impact of humor on behavioral intentions, such that the mediating role of competence is positive for men and negative for women.

In order to test the effects of gender, we used an experimental design with three conditions. Participants received an email from a new manager introducing him/herself by name (the manager’s name made gender explicit). The content of the email was introductory and also included a sentence declaring that time-off would not be granted in the following weeks because of exceptional work load. The three experimental conditions were created by toggling the ending of this email. The treatment group (humor condition group) received an ending that was an actual joke (related to time-off); the control group received an ending comprised of a general positive sentence (of the same length as the joke); the neutral group did not receive an additional ending to the basic message. The three improvements of Study 2 listed above, together with the experimental design, were intended to deepen the understanding of managerial humor, while testing the mixed-gender effects of sender and receiver.

### Method

#### Participants and Procedure

To detect a medium effect size of with 80% power (alpha = 0.05), G^∗^Power suggested we would need 65 participants for each group (Faul et al., 2009). As we had 4 groups, the sample size was acceptable. The final sample was comprised of 265 employees (64.50% women); the participants’ mean age was *M*_age_ = 28.00 (*SD*_age_ = 7.40). Approximately 57% held a BA degree and 11.30% held an MA degree or higher. The participants’ average weekly work hours were 33.30 (*SD*_hours_ = 15.30) and a total of 72.5% were in a non-management position. Study participation was voluntary. One MA student and three BA students recruited the participants as part of their degree requirements. The students distributed the questionnaires using their personal contacts, thus achieving heterogeneity of participants and jobs (Demerouti and Rispens, 2014). Data collectors directly described the objectives of the research to participants who were willing to take part in this experiment and sent them (a) an email that included a letter describing the purpose of the study and the assurance of anonymity of all responses; (b) a randomized email containing a message from the manager (see Appendix for email wording); and (c) an online questionnaire. The study received research ethics committee (institutional review board) approval.

#### Measurements

##### Perceived warmth and competence

To measure perceived warmth and competence, we used items developed by Fiske et al. (2002). Perceived warmth was measured using 4 items (sincere, friendly, tolerant, and warm). Perceived competence was measured using 5 items (skillful, confident, intelligent, competent, and efficient). All items were measured using a 7-point Likert scale – ranging from 1 (totally disagree) to 7 (totally agree). Cronbach’s alphas were (0.80) for perceived warmth and (0.86) for perceived competence.

##### Behavioral intentions

To measure behavioral intentions, we used two questions: “Would you like to continue working with this manager?” and “Would you like to meet the manager in person?” Participants responded on a 5-point Likert scale – ranging from 1 (do not want to) to 5 (definitely want to). The correlation between the two questions was 0.64 (*p* < 0.001). The measurement of behavioral outcome was the mean of the two questions.

##### Manipulation check

To assess whether the message was perceived as humorous, participants answered the following question: “Did you find the manager’s email amusing?” Participants responded on a 5-point scale – ranging from 1 (not amusing at all) to 5 (very amusing).

### Results

Descriptive statistics and intercorrelations are shown in Table 2.

**TABLE 2:** Descriptive statistics and intercorrelations – Study 2.

Variable	Mean	*SD*	1	2	3	4	5	
1. Humor	–	–	–	–	–	–	–	
2. Warmth	2.71	0.92	0.29***	(0.80)	–	–	–	
3. Competence	3.22	0.93	−0.03	0.58***	(0.86)	–	–	
4. Behavioral intentions	3.13	1.02	0.15*	0.60***	0.56***	(0.78)	–	
5. Manager’s gender	–	–	0.05	0.06	0.07	0.01	(–)	
6. Employee’s gender	–	–	0.07	0.06	0.04	−0.02	0.17*	(–)

*N* = 265. Reliability coefficients are displayed on the diagonal. Warmth = Employees’ perceived warmth of the manager; Competence = Employees’ perceived competence of the manager. Humor 0 = no, 1 = yes; *p < 0.05, and ***p < 0.001.

#### Manipulation Check

An analysis of variance (ANOVA) revealed a significant difference between the experimental and control groups [*F*(2,262) = 13.29, *p* < 0.001)]. *Post hoc* analysis (Scheffe) indicated that the participants in the humor condition were significantly more amused (*M* = 3.40, *SD* = 0.92) than the participants in the control group (*M* = 2.91, *SD* = 0.93) or the neutral group (*M* = 2.73, *SD* = 0.93).

#### Examining the Effects of Humor on Behavioral Intentions

In order to re-examine *H1*, predicting that a humorous comment in an email increases employees’ willingness to work and meet with their manager, a univariate analysis of variance (ANOVA) was conducted, while controlling for manager and employee gender. Results indicated that behavioral intentions were significantly different for the humor condition group as opposed to the control and neutral groups *F*(2,263) = 2.78, *p* < 0.05, partial η^2^ = 0.02. The *post hoc* test (Duncan) indicated that the humor condition group were more willing to work and meet with the manager (*M* = 3.31, *SD* = 0.99) as opposed to the neutral group (*M* = 2.99, *SD* = 0.87), thus supporting *H1*.

#### Examining the Effects of Perceived Warmth and Competence

In order to re-examine *H2a* and *H2b*, predicting that a humorous comment in an email increases employees’ perceptions of their manager’s warmth and competence, a multivariate analysis of variance (MANOVA) was conducted. As Box’s M (21.13) was significant (*p* = 0.002), indicating that there are significant differences between the covariance matrices, we proceeded to interpret Pillai’s Trace (Field, 2013). The MANOVA analysis indicated a significant effect *F* (2, 262) = 10.25, *p* < 0.001; Pillai’s Trace = 0.15, partial η^2^ = 0.07. Follow-up univariate ANOVAs indicated that warmth was significantly different for the humor condition group as opposed to the control and neutral groups *F*(2,262) = 12.26, *p* < 0.001, partial η^2^ = 0.09. The *post hoc* test (Scheffe) indicated that the humor condition group perceived the manager as warmer (*M* = 3.04, *SD* = 1.03), as opposed to the control group (*M* = 2.55, *SD* = 0.75) and the neutral group (*M* = 2.45, *SD* = 0.80), thus supporting *H2a*. However, there was no significant difference among the three groups regarding employees’ perceptions of the manager’s competence *F*(2,262 = 0.76, *p* = 0.47); thus, *H2b* was not supported.

#### Examining the Moderated Mediation Effect

We adopted Hayes (2017) procedure to test the moderated mediation model. We ran the analysis using the SPSS macro PROCESS 3.2, model 4 (for Hypothesis 3) and model 9 (for Hypothesis 4). As in Study 1, to reduce non-essential multicollinearity concerns (Enders and Tofighi, 2007), independent variables were mean-centered before computing product terms. To test Hypotheses 3a and 3b, a mediated regression analysis was conducted using Hayes (2017) PROCESS model 4 to test the mediation model. We tested the indirect effects using bootstrapping with 5,000 randomly generated samples. The results revealed that the indirect effect for warmth was significant (*B* = 0.22, *SE* = 0.06, 95% CI [0.12, 0.36]), supporting *H3a*. However, consistent with Study 1, the indirect effect of competence was not significant (*B* = −0.02, *SE* = 0.04, 95% CI [−0.11, 0.65]); thus, *H3b* was not supported.

Hypothesis 4 proposed that manager and employee gender would moderate the indirect effects of humor on behavioral intentions. These hypotheses were tested using Hayes (2017) PROCESS model 9 to test the moderated mediation model. As shown in Table 3, the predicted interaction – humor X manager’s gender – was not statistically significant (*B* = 0.26, *SE* = 0.24, *p* = 0.28). The predicted interaction – humor X employee’s gender – was statistically significant (*B* = −0.81, *SE* = 0.25, *p* < 0.001). A simple slope analysis indicated that humor positively affects male employees’ perceived competence of managers (*B* = 0.45, *SE* = 0.20, *p* = 0.02). However, humor negatively affects female employees’ perceived competence of managers (*B* = −0.32, *SE* = 0.14, *p* = 0.03).

**TABLE 3:** Moderation and mediation effects for humor.

Variable	*B*	*SE*	*t*	Boot 95% CI	*p*

Mediator variable model of warmth as a dependent variable					
Humor	0.55	0.11	4.90	0.33, 0.76	<0.001
Employee’s Gender	0.05	0.12	0.48	−0.17, 0.28	0.63
Manager’s gender	0.08	0.11	0.72	−0.14, 0.30	0.47
Humor X E.Gender	–0.31	0.24	–1.31	−0.78, 0.16	0.19
Humor X M.Gender	–0.31	0.23	–1.38	−0.76, 0.13	0.17

**Variable**	**B**	**SE**	**t**	**Boot 95% CI**	***p***

**Mediator variable model of competence as a dependent variable**					

Humor	0.04	0.12	–0.37	-0.27, 0.18	0.71
Employee’s Gender	0.11	0.12	0.90	−0.13, 0.34	0.37
Manager’s gender	–0.15	0.11	–1.27	−0.38, 0.08	0.20
Humor X E.Gender	–0.81	0.25	–3.29	−1.30, -0.33	<0.001
Humor X M.Gender	0.26	0.24	1.09	−0.21, 0.72	0.28

**Variable**	**B**	**SE**	***t***	**Boot 95% CI**	***P***

**Dependent variable model of the behavioral intentions**					

Humor	0.08	0.10	0.82	−0.11, 0.28	0.41
Warmth	0.42	0.06	6.51	0.29, 0.54	<0.001
Competence	0.34	0.06	5.63	0.22, 0.46	<0.001

*CI, confidence interval; E.Gender, Employee’s gender; M.Gender, Manager’s gender.*

**FIGURE 2:**
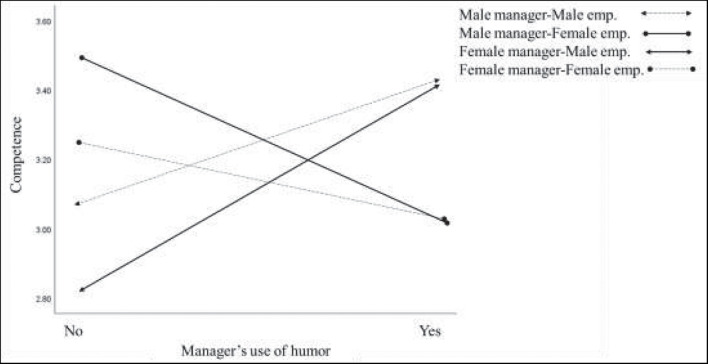
The interaction between manager’s use of humor and gender, on employees’ perception of manager’s competence. emp, employee.

When examining the moderated mediation hypothesis of manager’s gender, we looked at the index of the moderated mediation of warmth and competence. These indexes were not significant (effect = −0.13, 95% CI = [−0.35, 0.05]; effect = 0.09, 95% CI = [−0.06, 0.26], respectively). The moderated mediation hypothesis of employee’s gender was not significant for warmth (effect = −0.13, 95% CI = [−0.36, 0.07]), but it was significant for competence (effect = −0.28, 95% CI = [−0.51, −0.10]). Thus, *H4a* was supported for competence, but not for warmth. The findings show that employee gender moderated the mediating role of competence on the effects of humor in relation to behavioral intentions. Conditional indirect effects are shown in Table 4. Explicitly, the effect of female managers’ use of humor on competence was positive for male employees. However, the effect of male managers’ use of humor on competence was negative for female employees. The moderating effect is depicted graphically in Figure 2.

**TABLE 4:** Conditional indirect effects of humor on behavioral intentions via IM as a function of manager’s gender (W) and employee’s gender (Z).

		Warmth			Competence		
Manager’s gender (W)	Employee’s gender (Z)	Indirect effect	*SE*	Boot 95% CI	Indirect effect	*SE*	Boot 95% CI
Male	Male	0.39	0.12	0.18, 0.34	0.12	0.89	−0.52, 0.30
Male	Female	0.25	0.09	0.11, 0.45	–0.16	0.07	−0.33, −0.033
Female	Male	0.25	0.12	0.05, 0.52	0.20	0.92	0.04, 0.41
Female	Female	0.12	0.08	−0.02, 0.29	–0.75	0.59	−0.21, 0.03

*CI, Confidence interval.*

### Discussion

It has been established in the literature that liking someone depends on warmth, while respecting someone depends on competence (Fiske, 2018). Our findings in Study 2 support this notion, but take it even further. First, Study 2’s findings confirm the results of Study 1. In general, employees perceived a humorous manager as warmer, while his/her competence (as perceived by the employee) was not affected. Moreover, warmth mediated the relationship between humor and behavioral intentions, while competence did not. Study 2’s unique addition to these findings is the effect of gender as a moderator. Using a larger sample, we examined the effect of gender (both the manager’s as well as the employee’s), on the relationship of humor through IM on behavioral intentions. Our findings indicate that only employee gender interacted with this relationship. First, for men, humor positively affected employees’ perceptions of managers’ competence, while for women, it negatively affected this perception. The gender effect extended further when we considered the moderated-mediation findings. Both male and female employees, irrespective of the manager’s gender, perceived a humorous manager as warm. This result is consistent with the extant research on the primacy of warmth/communion (e.g., Abele and Wojciszke, 2007). However, when it came to employees’ perceptions of competence, the results showed a mixed-gender effect. Female employees perceived humorous male managers as less competent, while male employees perceived humorous female managers as more competent. Same-gender pairs did not show significant effects on perceptions of competence. We explain these findings using the theoretical ideas of how we perceive ourselves and others (Nehrlich et al., 2019), and social judgments (Abele et al., 2008). In line with these concepts, depending on the observer’s gender, people differ in the way they interpret humorous behavior (i.e., in terms of warmth or competence), and make a judgment accordingly. In terms of competence, if the actions are framed from the actor’s perspective and in warm–moral terms, it is interpreted from the observer’s perspective – which may explain the mixed-gender results.

## General Discussion

The current work documents significant associations linking the variables of humor, IM, and willingness to work with managers via email interface. We indicated that the use of humor boosts employees’ perceptions about a manager’s warmth, but does not impair competence-related perceptions, compared to non-humorous messages. In addition, both studies showed a robust and positive relationship between the use of humor and willingness to work with the manager via perceptions of warmth. Finally, in Study 2, we demonstrated that the receiver’s gender moderates the mediating role of competence on the association between accepting a humorous message from the manager and willingness to continue working with the manager.

Our findings offer a number of important theoretical contributions. First, our findings contribute to the cumulative knowledge on the impact of managerial humor on employee outcomes (Cooper, 2005; Yam et al., 2018). Managerial humor facilitates several beneficial key outcomes in the workplace because it is a relational currency that is valued by the employees (Kong et al., 2019). These qualities promote employees’ attitudes above and beyond other notable leader characteristics such as leader positive affect, the extraversion trait, and leadership consideration behaviors (Cooper K.M. et al., 2018). Research generally suggests that humorous individuals are perceived as more socially attractive (e.g., Greengross and Miller, 2011), and that humor is an effective method for gaining social visibility (Cooper, 2005). However, scholars know little about how managerial humor expression is conveyed, and how it affects employee behavior in organizations (Kong et al., 2019). In both studies, we found that by using humor, the manager can increase the perceived warmth generated by the humorous message. Because affection and warmth are common interpersonal goals for sustainable manger-employee relationships (Bolino et al., 2016), our research indicated that these factors can ultimately impact employees’ willingness to continue working with the manager. This hold true for both mundane, daily jobs, like motivating an employee to perform a boring, technical task; as well as for delivering email messages related to work directives (such as limiting vacations for the next month) – and this effect was not moderated by managers’ gender.

Second, our findings highlight the importance of managerial humor expression on IM processes, a relatively unexplored topic. Our study expands previous work that referred to humor as a useful strategy for achieving desirable impressions by reducing the social distance between communicators (Martin and Ford, 2018), and allowing interview candidates to deliver manipulated information more smoothly (Bitterly and Schweitzer, 2019). The current study indicates that managerial humorous email messages can boost the perception of warmth compared to non-humorous messages. However, we found that the use of humor did not affect the perception of competence. Managers often try to create a positive impression, but also struggle with the challenge of possibly reducing their status and authoritative power (Bitterly et al., 2017). It seems that humorous messages via email can help managers navigate these situations. It was suggested that social information, such as warmth, has more weight in evaluative judgments. From an evolutionary perspective, warmth as opposed to competence has a more profound effect on target behavioral outcomes, and elicits an approach-avoidance tendency towards the other person because it provides an immediate social cognition answer regarding whether the person is a friend or an enemy (Fiske et al., 2007). Therefore, cognitively, people are more sensitive to information related to warmth than to competence-related information (Fiske, 2018). Indeed, our results suggest that warmth perceptions are the most stable and positive quality a joke-teller can receive.

Third, while addressing previous calls (Kong et al., 2019), we make an important contribution to the humor-gender literature. The current study is the first to highlight an important relationship between the use of humor and the gender of both the manager and the employee. More specifically, we find that humor can have beneficial effects when the manager’s use of humor is directed to male employees, but may harm the IM of competence when communicated to females. We present several possible explanations for the negative impact of humor on female receivers. IM theories have already indicated that warmth and competence can be inversely linked, and that there is a warmth-competence tradeoff effect (compensation effect) in IM (Kervyn et al., 2009). However, these aspects are not necessarily compensated if the individual needs to make a cognitive decision (Kraft-Todd et al., 2017). Since females tend to respond more emotionally to given events (Fischer et al., 2018), it could explain why females in our study showed a compensating tradeoff between warmth and competence, while men did not. In addition, the humor/gender literature suggests that men and women may react differently to humor communications (Tosun et al., 2018), and the purpose of humor seems to shift between genders (Chan, 2016). Humor is usually regarded as a predominately masculine trait (Hooper et al., 2016); men use humor as a tool to reinforce social order and sustain the *status quo*. As opposed to men, women tend to use humor to build social affiliation and to promote group solidarity (e.g., Hay, 2000; Hooper et al., 2016). Building on the superiority theory, we can argue that female employees might be more sensitive to the managerial use of humor, which can emphasize managerial role superiority, and therefore increase more negative evaluations towards managers (either male or female). Another theoretical explanation to the moderation finding focuses on humor and appropriateness. According to the Benign Violation Theory (McGraw and Warner, 2014), a norm violation occurs during humorous interaction. Based on the theory, Yam et al. (2018) suggested that managers’ use of humor signals an acceptability of norm violation, which leads to negative outcomes such as employee deviance. Humorous norm violations may have other negative impacts; for example, sexist jokes may lead others to perceive sexism as more acceptable (e.g., Ford, 2000). Therefore, interactions with a humorous leader may encourage employees to break company rules, which might lead to boundary confusion (Li et al., 2019). It is possible that women employees are more sensitive to managerial norm violation behavior through the use of humor, and might be worried if the relationship with their manager becomes too informal or less serious. This is because such behaviors might lead other employees to form an organizational perception of norm violation acceptability, which can ultimately harm the organizational climate. Finally, as email is the most common workplace communication channel (Machili et al., 2019), the findings highlight the importance of humor in email communication. As contextual cues play an increasingly dominant role in our lives (Pelled et al., 2017), including our workplace, there is a growing need to understand the “rules of the game” of this growing new-media, text-based literacy. The current study explains the importance of CMC in relation to the ongoing conduct of employee-manager interaction. Our findings in Studies 1 and 2 illustrate how humor used by managers and its effect on IM can be manifested in a CMC and replicate results, which up until now, have only been observed in experiments through FtF communication. Although individuals tend to share more task-oriented and depersonalized information in their emails, as opposed to FtF interactions (e.g., Ebner, 2011), it seems that using humor may enrich the email communication channel, and IM strategies can account for this process as well. Our findings support the assumptions of SIP theory, suggesting that the need or desire to communicate relational information is no less important in CMC conversations than it is in FtF communication (Walther, 1992; Hancock, 2004). We indicated that the use of humor can be perceived as a complementary mechanism for relational information. We then extended this common practice and tested its effect in the workplace.

### Theoretical Implications

Managerial humor is an affective managerial tool; however, the literature on managerial humor “appears rather fragmented and much empirical research has notable shortcomings” (Kong et al., 2019, p. 4). Kong et al., in their meta-analysis review, suggested that the next step for future explorations on the role of leadership humor should focus on the role of managerial humor expression because it was a better predictor of employee attitudes and behaviors, as opposed to the managerial humor trait. They suggested that managerial humor expression creates a strong situation in which behaviors are directed by the context, regardless of a leader’s humorous tendency. Therefore, our studies shed important light on managerial and leader humor research by identifying the impact of leader humor expression on both employees’ perceptions (IM) and employee behavioral intentions to develop future interactions with the manager. Previous work on this topic mainly addressed the relationships between leadership humor expression, as measured by self-reported scales, and employees’ intentions to work in the organization. However, prior research provides only a very limited understanding of the relationship and limited empirical evidence (Kong et al., 2019). Our study contributes both theoretically and empirically to the topic by introducing both mediators and moderators to the associations between managerial humor expression and employee intentional behaviors. Specifically, Study 2’s findings provide promising directions for further development in the field. By emphasizing the moderating role of gender in these mediating paths, our research responds to the call for studies that document the underlying mechanisms of gender on leadership humor mechanisms (Mesmer-Magnus et al., 2018; Kong et al., 2019). Our research might also contribute to the methodological knowledge on managerial humor expression because it overcomes some of the limitations of the vast majority of these studies, including cross-sectional data and single-waved studies which are susceptible to common method/source bias.

Our studies also contribute to humor research by providing evidence for the applicability of humor in an e mail correspondence context. This is an important contribution because humor research within the realm of leadership humor expression has primarily focused on the use of humor in FtF. Therefore, the current study broadens our understanding of how humor operates in a non-traditional approach. The present research also contributes to the humor literature, which suggests the importance of using humor that is relevant to the message context, such as humorously written replies to customer complaints (Shin and Larson, 2020). Our work also provides insight into the way email correspondence uses humor, as an interpersonal resource for leaders to influence followers. Humor via email produces beneficial employee outcomes, such as managers’ positive impression management and employees’ enhanced willingness to continue working with the manager. While previous studies suggested that managers who use humor are perceived more favorably by their employees (i.e., Messmer, 2012), this research did not address written communication, but primarily FtF interactions that were measured via employees’ self-report. This is a critical gap in the available research because FtF interactions provide a very different medium for communication immediacy than CMC contexts (Schulze et al., 2017). The current work is the first to examine managers’ humorous statements in email communication. Our investigation will examine how adding a humorous statement in a manager’s email sent to employees affects employees’ perceived impression of the manager and their subsequent behavioral intentions.

### Practical Implications

Our study provides important practical implications for practitioners and managers. Humor can be a functional tool in the hands of the manager and may serve to strengthen employee-manager social exchange interactions (Cooper K.M. et al., 2018), reinforce followers’ behaviors and attitudes towards the organization, and improve organizational effectiveness (Mesmer-Magnus et al., 2012; Kong et al., 2019). Generally speaking, our findings suggest that an effective way to increase employees’ behavioral intention is by improving employee perceptions about their manager. Developing leader humor is one approach to achieving this goal. However, our results revealed that these effects are different among men and women. Since “you never get a second chance to make a first impression,” employees and managers at all organizational levels should recognize the importance of how their initial behavior is perceived at the beginning of their interaction. In applying the integrative social exchange theory to leadership humor (Cooper K.M. et al., 2018), we suggest that beginning with an initial interaction with the employee provides the first glimpses of warmth and competence impressions toward the manager, which facilitate employees’ willingness to form positive subsequent interactions with their manager. As we have proposed, there are strong theoretical reasons to expect humorous management to influence the fundamentals underlying employee outcomes. Humorous leaders can motivate their employees to “look on the bright side of things, redirect their employees away from the negative and instead focus on the positive, and on available opportunities (Li et al., 2019).

Managers may incorporate some humorous messages in several organizational announcements such as when introducing the organizational vision. They might even include some playfulness and humor in relation to cultural values such as: “don’t take yourself too seriously,” or “don’t forget to laugh today” (see Mesmer-Magnus et al., 2018 p. 707).

In addition, in the organizational setting, emails are routinely seen as providing a more convenient level of professionalism, and facilitating decision making and day-to-day work relationships (Derks and Bakker, 2010). Studies show there is a growing interest in the role that the language of email plays in university life and in business use (Levin and Kurtzberg, 2020). The use of humor in this formal, business-oriented genre is different than the use of humor in other informal electronic communication platforms (such as social networks). Humor is not frequently used in written interaction in the workplace; however, our work suggest that some expression of humor may be beneficial – even in a professional email correspondence. Therefore, we conclude that when managers add humor to their emails, they can always “score points” on warmth, and sometimes also on competence (although, as we have shown, competence perception is gender-sensitive and is probably content-dependent as well). Humor in email is more effective when distributed to receivers with shared interests and within a community (just like in work emails). In these situations, it is not difficult for managers to creatively assimilate a joke into their own discourse (Hübler and Bell, 2003). Doing so in emails (?) is even “safer” than in FtF communication, since it allows the manager to carefully control the quality of the writing and evade the pressure of an immediate oral response and/or embarrassment (ibid). All in all, since managerial humor is personally “safer” and relatively simple to use, and as it can increase employees’ perception of the manager’s warmth and sometimes competence, it is possibly the most inexpensive, effective, and accessible tool that managers have. With the continued growth of email use (Clement, 2019), along with other written media such as Twitter and LinkedIn (Smith, 2013), the importance of being able to manage both individual and organizational impressions online has become ever more apparent.

### Limitations and Future Directions

Although there are several strengths in the current research design – such as our effort to experimentally examine humor, the focus on email humor, and replicating the findings across two distinct situations and different samples – several limitations warrant further discussion and future research. First, the use of self-reports for the mediator and the outcome could be a cause of concern regarding CMV bias (Podsakoff et al., 2012), but this concern is reduced for several reasons. First, CMV is minimized due to the experimental design, which implies causality. In addition, we applied Podsakoff et al. (2012) method to diminish the impact of common method bias by ensuring participants’ anonymity. Therefore, overall, it seems that CMV is less likely to be a major concern in our experimental study as opposed to field and survey samples. Secondly, in Study 1 we tested the relationship between leader humor expression and employee behavioral intention using a one-item scale. Previous work used similar one-item measurements to examine whether managers and employees make an effort to develop their relationship (Maslyn and Uhl-Bien, 2001) that can result in equally valid measurement (Gardner et al., 1998). However, we acknowledge that a better and more extended measurement of behavioral intentions is needed, and that future studies will benefit from a rigorous validation of the measurement. We also acknowledge the notion that intentions do not always translate into behaviors. Thus, our results should be further validated in a future study. In addition, in both of our studies, we used positive affiliative humor and indicated that it influenced perceivers’ perceptions. Sporadic attention has been paid to managerial negative and antisocial humor expression (e.g., Yam et al., 2018). Future work should extend our investigation to explore how the use of other types of humor – such as aggressive or self-defeating humorous behaviors (Martin et al., 2003) –alters employees’ perceptions. In addition, given that humor is culturally bound (e.g., Kalliny et al., 2006), culture can be another promising moderator for the effects of leader humor, despite the limited evidence existing in regard to this issue. Business sector type (such as service-based versus non-service-based businesses) might also be another promising moderator for the role of managerial humor (Bompar et al., 2018). Future research would do well to study cultural influences on impression effectiveness by exploring behaviors mediating impression success among other cultural, national, or ethnic backgrounds. Finally, the experimental set-up may limit the study’s external validity, and the current research is limited to the variables examined. We encourage other investigators to focus on the use of humor behaviors in the workplace, along with impression management and willingness to work with the manager, in order to provide a generalization that can include experimental studies in the real world.

We call upon future research to expand the existing understanding of the associations linking humorous interactions, IM, and potential behavioral outcomes. Specifically, several important questions for future research remain unanswered. For example, how can managers effectively implement the use of humor in the workplace? Are there better and worse implementations of humor? And are there different managerial motivations for the use of humor? For example, managers can choose to use humor to achieve certain goals such as: identification with the target audience, clarification of ideas in memorable ways, enforcement of norms, or mitigating stressful events (Holmes et al., 2019). Future studies may wish to clarify whether the message content and the underlying goals of using humor could impact the manager’s beneficial use of humor. In addition, although humor appears to have positive consequences, there is also a downside to using humor at work. There is some evidence indicating that unsuccessful attempts to be humorous can reduce status (Bitterly et al., 2017), and that the violation of norms often accompanying humor leads to higher levels of employee deviance (Yam et al., 2018). The purpose of future research could focus on understanding when humor can be beneficial or detrimental in email correspondence. It may also be interesting to examine the negative impact of humor on the evaluation of competence among female audiences. In fact, one of the greatest challenges faced by working women is that they are undervalued in terms of their competence, as the general stereotype of women – being inherently warm and nurturing – is inconsistent with the drivers of career competence (Eagly and Karau, 2002). Future research should also explore other potential mediators of the relationship between humor and female behavioral outcomes.

We also call for future work to distinguish between the effects of humorous attempts and the successful use of humor. If members of an audience fail to find a humorous message conveyed in an email funny (meaning, the attempt at humor backfires and is perceived in a negative way), they may infer that the email is insulting, thereby negatively influencing their ability to accept the message.

Finally, we focused on individual-level outcomes for the joke-teller as well as the audience. However, humor is likely to have a significant impact at the dyadic, group, and organizational levels as well (Cooper, 2005). Just as the two universal dimensions of impression management – warmth and competence – can operate at both the individual level and the group level (Fiske et al., 2007), it is recommended that future studies will expand the current findings to include team organizational-level analyses.

### Final Conclusion

Humor is a managerial tool that can influence employees’ perceptions, intentions, and behaviors. Across our studies, the use of humor affected employees’ attitudes toward a manager’s warmth and competence, eventually influencing the employee’s behavioral intentions. Though emails pervade our everyday communication at the workplace, we still have much to learn about the key role managerial humor plays in how employees form beliefs and impressions of their supervisor and respond to them. The good news is that, armed with knowledge and awareness, managers can take on these challenges and even turn the use of humor to their advantage. Given the anticipated increase in email usage in the workplace (Clement, 2019), the sooner managers and employees start refining their skills and awareness in this area, the better.

## Data Availability Statement

The raw data supporting the conclusions of this article will be made available by the authors, without undue reservation.

## Ethics Statement

The studies involving human participants were reviewed and approved by Ariel Ethics Committee for research in human subjects. The patients/participants provided their written informed consent to participate in this study.

## Author Contributions

YB-I and AR contributed to the formation of ideas, methodology design, data collection, statistical analysis, and writing of the final manuscript. Both authors contributed to the article and approved the submitted version.

## Conflict of Interest

The authors declare that the research was conducted in the absence of any commercial or financial relationships that could be construed as a potential conflict of interest.

## Publisher’s Note

All claims expressed in this article are solely those of the authors and do not necessarily represent those of their affiliated organizations, or those of the publisher, the editors and the reviewers. Any product that may be evaluated in this article, or claim that may be made by its manufacturer, is not guaranteed or endorsed by the publisher.

## Appendix

### Emails Used in Study 1

#### Email Without a Joke – Neutral Version

“Hello. I’m the manager in charge of the department. Attached to this email is a list of the department’s expense items for the past month. Please choose which item is unnecessary in your eyes and should be removed from the list. Please email me the name of the chosen item. Thanks.”

#### Email Without a Joke – Control Version

Same as above, but with the addition of this sentence at the beginning of the email: “Since each department is required to present its expenses to the management each month, it is crucial that we cut our expenses when we need to report them to the people responsible for the budget.”

#### Email With Joke – Experimental Version

Same as in the neutral version, but with the addition of this sentence: “You should seriously consider whether or not to choose the superglue, since it will be difficult to remove from the list after 3 s.”

The list sent to the participants included 13 items, one of which was 20 tubes of super-glue. This information is important to understand the joke.

### Emails Used in Study 2

#### The Email From a Manager Without a Joke

Hi,

My name is Ron, and I will be starting this week as the new team manager.

Because of the new challenges facing us in the near future, I will unfortunately not be able to approve any days off over the coming month until things stabilize for our team.

Wishing us all a successful year.

Ron

#### The Email From a Manager With a Joke

Hi,

My name is Ron, and I will be starting this week as the new team manager.

Because of the new challenges facing us in the near future, I will unfortunately not be able to approve any days off over the coming month until things stabilize for our team.

Before I sign off, I want to share something with you: Two employees ask for some time off. One employee jumps on his desk and hangs his head upside-down. The manager runs to him and asks, “What are you doing?” The employee replies, “I’m a lamp.” The manager says, “I think you should take a few days off and pull yourself together.” The employee happily gets up and leaves. Immediately, the other employee gets up and packs his things. The manager asks him, “Where are YOU going?” He replies, “Home. How can we work without light?”

Wishing us all a successful year.

Ron
